# Impact of recipient body mass index on heart transplantation outcomes

**DOI:** 10.3389/fcvm.2025.1573589

**Published:** 2025-09-25

**Authors:** Matiullah Masroor, Chen Jiang, Tixiusi Xiong, Yixuan Wang, Nianguo Dong

**Affiliations:** ^1^ Department of Cardiovascular Surgery, Wuhan Union Hospital, Tongji Medical College, Huazhong University of Science and Technology, Wuhan, China; ^2^ Department of Cardiothoracic and Vascular Surgery, Amiri Medical Complex, Kabul, Afghanistan

**Keywords:** heart transplantation, recipient BMI, survival outcomes, post-transplantation outcomes, obesity

## Abstract

**Objective:**

Obesity is one of the common risk factors for heart failure. Heart transplantation (HTx) is a gold standard treatment option for end-stage heart failure. The relationship between obesity and HTx outcomes is not very clear. This study aims to investigate the impact of recipient BMI on heart transplantation outcomes.

**Methods:**

From 2012–2021, 821 patients underwent HTx in our center. Patients with age less than 18 years, multiorgan transplantation, re-transplantation, and missing recipient BMI data were excluded. The remaining 694 patients were divided into four BMI categories based on the recipient's BMI value. BMI < 18.5 kg/m^2^ (*n* = 70), BMI 18.5–24.99 kg/m^2^ (*n* = 432), BMI 25–29.99 kg/m^2^ (*n* = 156), and BMI ≥ 30 kg/m^2^ (*n* = 36). Analysis of variance and chi-square test with *post hoc* test according to the types of variables was performed to find differences among the groups. Kaplan–Meier analysis with a Log-rank test was performed for the survival analysis. Cox regression analysis was performed to adjust for confounders and see the effect of variables on mortality.

**Results:**

Some preoperative variables, such as recipient gender and diabetes status, were statistically significantly different between the groups. However, there was no significant difference in the postoperative outcomes except for high intra-aortic balloon pump (IABP) use in the BMI ≥ 30 kg/m^2^ group based on unadjusted analysis. The Kaplan–Meier survival analysis showed no short or long-term survival difference between the groups. The 5-year survival was 75%, 70%, 71%, and 83% in underweight, normal weight, overweight, and obese recipient BMI groups, respectively. Recipient BMI was not associated with follow-up mortality on multivariable analysis. Preoperative IABP use, history of chronic kidney disease, and recipient age were the independent risk factors for long-term mortality. The risk of mortality was five times higher in patients with preoperative IABP use and two times higher in patients with a history of CKD. A one-year increase in recipient age was associated with a 3.4% increase in mortality risk.

**Conclusion:**

The recipient BMI did not significantly impact post-transplantation survival after multivariable adjustment. However, unadjusted analyses showed comparable non-survival outcomes across BMI groups. Our study suggests that selected underweight and class I obese patients may undergo heart transplantation without increased risk of post-transplantation mortality.

## Introduction

Heart failure is affecting over 64 million people worldwide (1). The life expectancy of patients with end-stage heart failure is from less than 6 months to 1 year (2). Less than one out of five people who suffered from acute heart failure lived up to 10 years in New Zealand and Australia (3). There are multiple factors involved in the development of heart failure, and obesity is one of the prominent risk factors (4–7). More than 71% of the US population older than 20 years is overweight or obese, with more than 41% having obesity (8). It is estimated that more than 50% of the world population will be overweight or obese by 2035, with 25% having obesity (8).

Kenchaiah et al. (4) investigated the relationship between BMI and heart failure in 5,881 individuals in the Framingham Heart Study. They found that the risk of heart failure increased by 5% for men and by 7% for women, with an increment of 1 in BMI. The risk of heart failure was twice as high for an obese individual compared to a normal body weight in their study (4). Interestingly, the outcomes of heart failure patients who are overweight or class I obese are better than the underweight or normal-weight heart failure patients, an idea which is termed the “obesity paradox” (9–13). Heart transplantation is considered the optimal treatment option for end-stage heart failure (14).

The International Society for Heart and Lung Transplantation (ISHLT) 2006 guidelines for HTx candidacy recommend a BMI of less than 30 kg/m^2^ or an ideal body weight percentage of less than 140% before transplantation (15). The BMI > 35 kg/m^2^ in the 2006 and 2016 guidelines updates was a strong contraindication for HTx candidacy (15, 16). A BMI higher than the threshold can result in increased risk of infection, allograft vasculopathy, heart failure, hypertension, hyperlipidemia, diabetes mellitus, risk associated with HTx, slow wound healing, and increased post-transplantation short- and long-term mortality (15–18).

However, the relationship between BMI and HTx outcomes remains controversial. A study divided patients based on a BMI cutoff value of 25 kg/m^2^. Overweight patients with a BMI > 25 kg/m^2^ were associated with improved survival after HTx, but those patients also suffered more acute rejection, graft vasculopathy, and steroid-induced diabetes mellitus, while non-overweight patients had worse survival, renal dysfunction, more re-hospitalization, and lymphoma (19). Some studies have found no association between BMI and post-transplantation outcomes. Carrier et al., (20) in a multivariable logistic analysis, found that BMI did not affect postoperative one-month mortality. Another study also concluded that baseline BMI did not affect survival (21). A study conducted by Russo et al. (22) divided patients into underweight (BMI < 18.5 kg/m^2^), normal weight (BMI 18.5–24.99 kg/m^2^), overweight (BMI 25–29.99 kg/m^2^), Class I obese (BMI 30–34.99 kg/m^2^), and Class II/III obese (BMI ≥ 35 kg/m^2^) categories. On multivariable Cox regression analysis, they found that overweight and class I obesity were not significantly associated with decreased survival after HTx. However, the underweight and obese class II/III categories had significantly lower post-transplantation survival. On the other hand, some studies have found that higher BMI is associated with worse post-transplantation outcomes (23, 24).

The discrepancies of BMI's relationship with improved outcomes, no effect on outcomes, or worse outcomes after heart transplantation warrant further research. The objective of this study is to investigate whether the recipient's BMI impacts heart transplantation outcomes, including short- and long-term survival.

## Methods

### Study population

**Figure 1:**
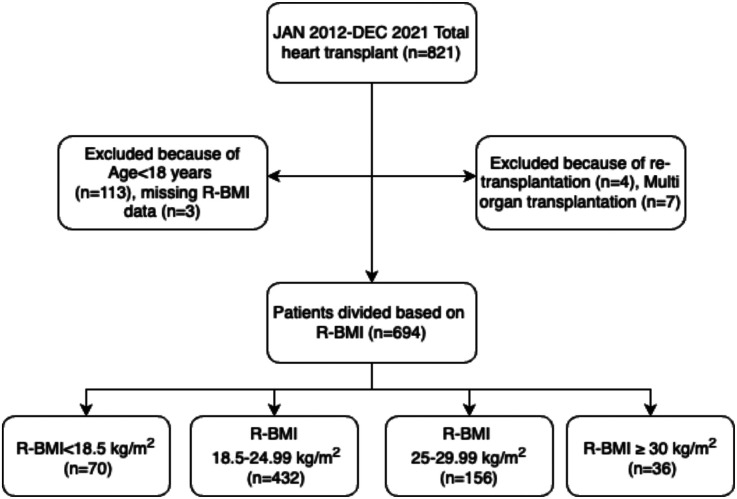
The flowchart of the study design.

Between January 2012 and December 2021, 821 heart transplant surgeries were performed at our institution. However, after excluding patients under the age of 18 years (*n* = 113), patients with missing recipient BMI data (*n* = 3), re-transplantation (*n* = 4), and multi-organ transplant (*n* = 7), a final sample of 694 patients divided into four groups based on recipient BMI was included in the analysis. The four groups were: underweight group (*n* = 70) (BMI < 18.5 kg/m^2)^, normal weight group (*n* = 432) (BMI 18.5–24.99 kg/m^2^), overweight group (*n* = 156) (BMI 25–29.99 kg/m^2^), and obese group (*n* = 36) (BMI ≥ 30 kg/m^2^). The study design is presented in Figure 1.

### Data collection and patients' follow-up

The data for heart transplant recipients were collected from patient records, follow-up hospital visits, and telephone contact with patients or relatives. The data includes demographic information, preoperative clinical information, surgical procedure information, and postoperative outcomes. Follow-up was done consistently based on hospital protocols. All donor grafts in this study were procured from brain-dead donors and allocated by the China Organ Transplant Response System. In compliance with the Declaration of Istanbul and international ethical standards, no grafts from an executed prisoner were utilized. The clinical and research activities are consistent with the principles of the Declaration of Helsinki and the Declaration of Istanbul.

Donor heart procurement and preservation methods, and Immunosuppression therapy were the same as described previously (25).

### Statistical analysis

Statistical analysis was conducted using SPSS version 29.0.1.0. Continuous variables were presented as means ± standard deviations. The means of continuous variables were compared by analysis of variance (ANOVA) to find the difference between the groups. Levene's test of homogeneity of variance was performed for data homogeneity. The ANOVA results were used when the variances were homogeneous, and the Welch's ANOVA was used when the variances were heterogeneous. Hochberg's GT2 test was used for *post hoc* analysis (which is the best fit for our data of different sample sizes in the groups) when homogenous variances were observed, and Games-Howell's test was used for unequal variances. The categorical variables were presented as frequencies and percentages. The chi-square test was used for categorical variables to compare different groups, and a pairwise chi-square for the *post hoc* analysis. The Bonferroni correction was applied by dividing the alpha value of 0.05 by the number of comparisons performed to achieve the new significant alpha value. Survival rates were estimated using the Kaplan–Meier method, with survival curves plotted for different BMI groups. The Log-Rank test was used to compare survival between these groups. An alpha value less than 0.05 was considered statistically significant. To explore the effect of different variables on follow-up mortality and adjust for confounders, univariable and multivariable Cox regression analyses were performed. Variables in the multivariable analysis were selected based on HR and clinical significance with the backward stepwise likelihood ratio method.

## Results

### Baseline and clinical characteristics

We compared demographic and baseline clinical characteristics between the different recipient BMI groups. Because the groups were created based on recipient BMI, the recipient weight, height, and BMI were also statistically significantly different between the groups. There were significant differences in the recipient gender, diagnosis, history of diabetes, chronic liver disease, and donor BMI between the groups. Other than that, there was no significant difference in all other preoperative variables, such as recipient and donor age, cold ischemic time, associated comorbidities, peripheral vascular disease, preoperative mechanical circulatory support use, preoperative EF, donor cause of death, etc., as given in Table 1.

**Table 1:** Comparison of demographics and baseline clinical characteristics of the different recipient BMI-based groups.

Variables	BMI < 18.5 Kg/m^2^ (*n* = 70)	BMI 18.5–24.99 Kg/m^2^ (*n* = 432)	BMI 25–29.99 Kg/m^2^ (*n* = 156)	BMI ≥ 30 Kg/m^2^ (*n* = 36)	*p*-value
Recipient age (years)	42.8 ± 16.1	48.2 ± 12.2	48.2 ± 11.2	46.1 ± 11.2	.051
Recipient gender (male)	40 (57.1%)	335 (77.5%)	142 (91.0%)	31 (86.1%)	**<** **.** **001**
Recipient weight (Kg)	46.6 ± 6.1	61.4 ± 7.7	77.2 ± 6.8	94.4 ± 15.0	<.001
Recipient height (cm)	163.8 ± 10.0	167.4 ± 7.0	169.7 ± 6.7	169.5 ± 8.0	<.001
Recipient BMI (Kg/ m^2^)	17.3 ± 1.0	21.8 ± 1.9	26.8 ± 1.3	32.7 ± 3.2	<.001
Diagnosis					**<**.**001**
NICM (%)	45 (64.3%)	275 (63.7%)	90 (57.7%)	31 (86.1%)	
ICM (%)	3 (4.3%)	87 (20.1%)	51 (32.7%)	4 (11.1%)	
VHD (%)	13 (18.6%)	45 (10.4%)	12 (7.7%)	0 (0%)	
CHD (%)	9 (12.9%)	14 (3.2%)	2 (1.3%)	0 (0%)	
Others (%)	0 (0%)	11 (2.5%)	1 (1.6%)	1 (2.8%)	
Ischemic time (min)	318 ± 107	321 ± 116	321 ± 104	313 ± 130	.980
Donor age (years)	37.5 ± 11.6	36.2 ± 11.2	35.2 ± 11.7	35.1 ± 10.21	.495
Donor gender (male)	59 (84.3%)	360 (85.7%)	135 (90.6%)	34 (97.1%)	.110
Donor weight (kg)	62.0 ± 10.3	63.2 ± 10.1	65.5 ± 10.1	69.2 ± 8.5	**<**.**001**
Donor height (cm)	168.0 ± 6.2	168.4 ± 5.8	169.1 ± 5.6	172.0 ± 3.5	.**002**
Donor BMI (kg/ m^2^)	21.9 ± 2.9	22.3 ± 2.9	22.8 ± 3.1	23.4 ± 2.7	.**036**
Hx of cardiac surgery (%)	21 (42.0%)	87 (30.4%)	28 (26.2%)	3 (15.0%)	.096
Hx of diabetes (%)	3 (7.7%)	43 (18.0%	31 (31.3%)	10 (52.6%)	**<**.**001**
Hx of CKD (%)	2 (3.6%)	18 (5.4%)	7 (5.3%)	2 (8.0%)	.873
Hx of CLD (%)	5 (8.9%)	26 (7.8%)	1 (0.8%)	2 (8.0%)	.**029**
HX of PVD (%)	3 (5.5%)	8 (2.4%)	5 (3.8%)	1 (4.0%)	.610
Preop IABP (%)	2 (3.4%)	8 (2.4%)	2 (1.5%)	1 (4%)	.797
Preop ECMO (%)	1 (1.7%)	8 (2.4%)	4 (3.0%)	1 (4.0%)	.913
Preop ventilation (%)	1 (1.7%)	15 (4.5%)	5 (3.8%)	1 (4.0%)	.805
Preop EF (%)	27.9 ± 11.8	28.4 ± 12.1	26.2 ± 8.6	27.8 ± 11.1	>.99
Cause of donor death					.527
TBI (%)	39 (58.2%)	258 (61.3%)	83 (55.3%)	11 (44.0%)	
CVD (%)	21 (31.3%)	132 (31.4%)	47 (31.3%)	13 (52.0%)	
Brain tumor (%)	3 (4.5%)	13 (3.1%)	7 (4.7%)	0 (0%)	
Anoxic brain death (%)	3 (4.5%)	14 (3.3))	10 (6.7%)	1(4.0%)	
Else (%)	1(1.5%)	4(1.0%)	3(2.0%)	0(0%)	

BMI, body mass index; ICM, ischemic cardiomyopathy; NICM, non-ischemic cardiomyopathy; VHD, valvular heart disease; CHD, congenital heart disease; CKD, chronic kidney disease; CLD, chronic liver disease; PVD, peripheral vascular disease; IABP, intra-aortic balloon pump; ECMO, extracorporeal membrane oxygenation; LVAD, left ventricular assist device; TBI, traumatic brain injury; CVD, cerebrovascular diseases.

The bold values indicated statistical significance.

In the *post-hoc* analysis of the variables that were statistically significantly different, each group was compared to the others, and the *p*-value is shown in Table 2. The percentages of these variables are listed in Table 1. Donor weight and height were significantly lower in the underweight and normal weight groups compared to the obese group. There was no significant difference in donor BMI among the groups according to the Hochberg GT2 test. Male recipients were fewer in the underweight group compared to all other groups. Additionally, male recipients were fewer in the normal weight group compared to the overweight group. Diabetic patients were fewer in the underweight and normal weight groups compared to the overweight and obese groups.

**Table 2:** *Post-hoc* analysis of the significant continuous and categorical variables and the probability values of each group compared to the other groups.

Variables	R-BMI < 18.5 kg/m^2^			R-BMI 18.5–24.99 kg/m^2^		R-BMI 25–29.99 kg/m^2^
	R-BMI 18.5–24.99 kg/m^2^	R-BMI 25–29.99 kg/m^2^	R-BMI ≥ 30 kg/m^2^	R-BMI 25–29.99 kg/m^2^	R-BMI ≥ 30 kg/m^2^	R-BMI ≥ 30 kg/m^2^
D-Height	.994	.611	**.** **004**	.618	.**002**	.050
D-Weight	.924	.083	.**003**	.075	.**004**	.267
D-BMI	.879	.199	.117	.379	.264	.911
R-Gender (male)	**<0**.**001**	**<0**.**001**	.**003**	**<0**.**001**	.232	.373
R-Diagnosis						
NICM	.919	.350	.018	.188	.007	.**001**
ICM	.**0013**	**<0**.**0001**	.180	.002	.189	.010
VHD	.048	.016	.006	.324	.042	.086
CHD	.**0003**	.**0002**	.025	.197	.273	.495
Else	.177	0.502	.161	.149	.933	.255
DM	.109	.**004**	**<0**.**001**	.**007**	**<0**.**001**	.074
CLD	.765	.**003**	.891	.**003**	.966	.015

The Bonferroni corrected significant alpha level for all categorical variables is.008, except for diagnosis, which is .0017. The significance level for continuous variables is .05.

The bold values indicated statistical significance.

### Postoperative outcomes

The post-operative outcomes of the four recipient BMI groups were not statistically significantly different except for IAPB usage, as shown in Table 3. The use of postoperative IABP was higher in the obese group (58.3%) and was significantly different than other groups during the *post hoc* analysis (*p* = 0.002; after Bonferroni correction, the alpha value was set at 0.008). Other postoperative outcomes, such as acute rejection, mechanical ventilatory support, ICU stay, respiratory, renal, or neurological complications, and use of mechanical circulatory support devices, were not statistically different among the groups, even though the diabetic patients were more in the overweight and obese category compared to the normal weight category. These results should be interpreted with caution as these can be affected by the differences in the baseline variables.

**Table 3:** Comparison of postoperative outcomes of different recipient BMI-based groups.

Variables	BMI < 18.5 (*n* = 70)	BMI 18.5–24.99 (*n* = 432)	BMI 25–29.99 (*n* = 156)	BMI ≥ 30 (*n* = 36)	*p*-value
CPB time (min)	118 ± 37	119 ± 61	117 ± 38	127 ± 51	.340
Cross-clamp time (min)	33 ± 12	33 ± 11	33 ± 9	32 ± 6	.097
Surgery time (min)	256 ± 66	276 ± 97	270 ± 103	286 ± 88	.958
Postop ventilation (hrs)	55.5 ± 71.4	83.4 ± 203	74.0 ± 177	66.8 ± 72	.656
ICU stay (days)	10.3 ± 7.6	10.8 ± 11.0	11.3 ± 9.9	11.4 ± 6.6	.893
Acute rejection (%)	2 (3.4%)	5 (1.5%)	3 (2.3%)	0 (0.0%)	.639
Respiratory complication (%)	38 (65.5%)	208 (61.9%)	82 (61.7%)	17 (68.0%)	.887
Neurological complication (%)	5 (8.8%)	26 (7.9%)	6 (4.5%)	2 (8.0%)	.602
Renal complication (%)	11 (19.3%)	58 (17.6%)	23 (17.4%)	5 (20.0%)	.979
Septic shock (%)	1 (2.2%)	15 (6.0%	1 (1.0%)	1 (4.8%)	.167
Blood culture +ve (%)	10 (18.5%)	40 (13.2%)	18 (14.3%)	4 (16.7%)	.748
Sputum culture +ve (%)	27 (49.1%)	164 (51.7%)	75 (58.6%)	14 (56.0%)	.528
Postop EF (%)	66.2 ± 6.0	65.0 ± 6.0	64.5 ± 7.9	65.7 ± 5.1	.277
Postop IABP (%)	20 (28.6%)	143 (33.1%)	60 (38.5%)	21 (58.3%)	**.** **010**
Postop ECMO (%)	5 (7.1%)	29 (6.7%)	9 (5.8%)	4 (11.1%)	.720
Postop CRRT (%)	10 (15.2%)	65 (15.3%)	25 (16.0%)	5 (13.9%)	.990
Postop hospital stay (days)	36.6 ± 17.1	37.8 ± 21.7	41.3 ± 21.7	41.9 ± 24.3	.217

CPB, cardiopulmonary bypass; BMI, body mass index; ICU, intensive care unit; RBC, red blood cell; EF, ejection fraction; IABP, intra-aortic balloon pump; ECMO, extracorporeal membrane oxygenation; CRRT, continuous renal replacement therapy.

The bold values indicated statistical significance.

### Survival analysis

**Figure 2:**
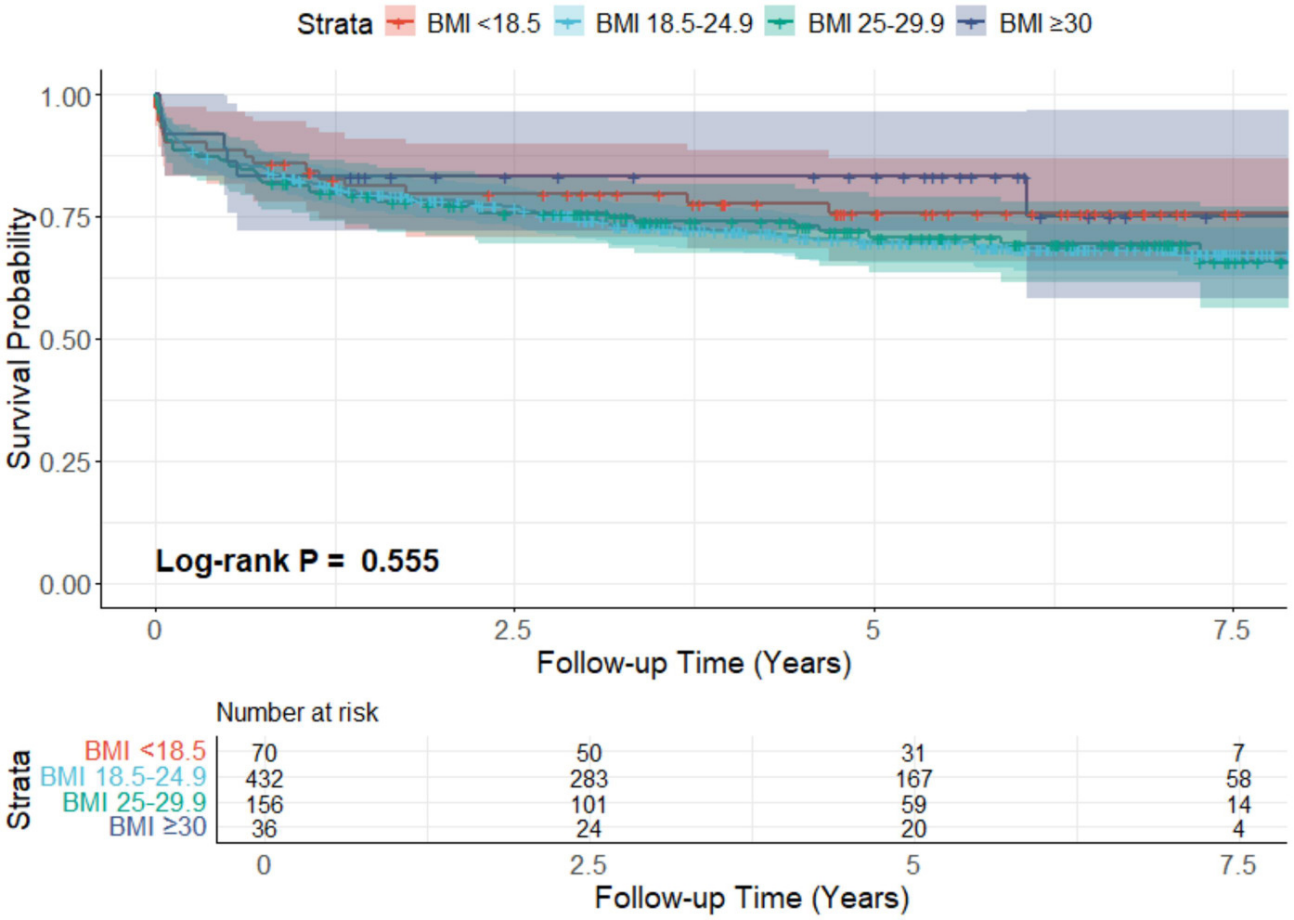
The KM survival curves of the four different recipient BMI-based groups.

The overall mean survival time was 93.2 ± 2.2 months, while restricted mean survival time was 61.9 ± 1.7 months. The survival rate on the Kaplan–Meier survival curve of the underweight group at 1,3,5, and 7 years was 85.7% ± 4.2%, 79.6% ± 4.9%, 75.7% ± 5.4%, and 75.7% ± 5.4%, respectively. For the normal-weight group, 1-year survival was 82.6% ± 1.8%, 3-year survival was 74.3% ± 2.2%, 5-year survival was 69.8% ± 2.4%, and 7-year survival was 68.3% ± 2.5%. The survival for the overweight group, at 1,3,5, and 7 years, was 81.4% ± 3.1%, 75.8% ± 3.5%, 70.8% ± 3.9%, and 69.3% ± 4.1% respectively. For the obese group, 1,3, and 5-year survival was 83.3% ± 6.2%, and 7-year survival was 75.0% ± 9.7%. The log-rank test result was (*p* = 0.555), which shows no difference in survival between the groups. The KM survival curve is presented in Figure 2.

### Cox regression analysis

To investigate the impact of preoperative variables on the follow-up mortality and ensure that the comparable outcomes between the groups were not associated with preoperative variables. We performed univariable and multivariable Cox regression analysis. The risk factors associated with follow-up mortality in univariable analysis were recipient and donor age, history of CKD, preoperative mechanical ventilation, and preoperative IABP and ECMO use. Interestingly, male recipient gender was associated with a 30.2% decrease in follow-up mortality compared to female recipients. Underweight, overweight, and obese recipients had no increased risk of death compared to the normal weight recipients. The results of the univariable analysis are shown in Table 4.

**Table 4:** Univariable Cox regression analysis for follow-up mortality.

Variables	HR	95% CI	*p*-value
Recipient age (years)	1.028	1.015–1.041	**<** **.** **001**
Recipient gender (male)	.698	.506–0.964	.**029**
Recipient BMI			.560
BMI 18.5–24.99	Ref	Ref	Ref
BMI <18.5	.775	.460–1.304	.336
BMI 25–29.99	.993	.704–1.400	.967
BMI ≥ 30	.650	.303–1.391	.267
Diagnosis			<.001
NICM	Ref	Ref	Ref
ICM	1.255	.886–1.777	.2
VHD	.685	.386–1.216	.196
CHD	1.227	.572–2.633	.599
Others	6.161	3.454–10.990	<.001
Ischemic time (min)	1.001	1,000–1.002	.102
Donor age (years)	1.018	1.005–1.031	.**006**
Donor gender (male)	.795	.528–1.196	.271
Donor weight (kg)	1.003	.989–1.017	.658
Donor height (cm)	.999	.974–1.024	.918
Donor BMI	1.023	.975–1.073	.353
Hx of cardiac surgery	1.294	.898–1.865	.166
Hx of Diabetes mellitus	1.523	.989–2.343	.056
Hx of CKD	2.24	1.312–3.823	.**003**
Hx of CLD	.573	.253–1.297	.182
HX of PVD	1.156	.474–2.820	.75
HX of IABP	3.747	1.830–7.670	**<**.**001**
Hx of ECMO	3.036	1.428–6.569	.**004**
Preop ventilation	2.523	1.325–4.803	.**005**
Preop EF < 25	.750	.557–1.010	.058
Cause of donor death			.796
TBI	Ref	Ref	Ref
CVD	1.205	.882–1.646	.241
Brain tumor	.961	.422–2.188	.924
Anoxic brain death	1.220	.594–2.505	.588
Else	.863	.213–3.498	.837

The bold values indicated statistical significance.

Nine variables, recipient and donor age, recipient gender, history of CKD, preoperative mechanical ventilation, IABP, and ECMO use, diabetes mellitus, and recipient BMI groups based on hazard ratios and clinical importance, were included in multivariable analysis. The recipient's BMI as a categorical variable, in addition to the other variables, was included in the multivariable model to observe its effect on mortality after adjusting for the confounding effect of other variables. Recipient age, history of CKD, and preoperative IABP use retained their statistical significance. The protective effect of the male recipient observed during univariable analysis was lost during multivariable analysis. The multivariable Cox regression analysis is given in Table 5.

**Table 5:** Multivariable Cox regression analysis for follow-up mortality.

Variables	HR	95% CI	*p*-value
Recipient age (yrs)	1.034	1.016–1.052	**<** **.** **001**
Recipient gender (male)	.791	.510–1.226	.295
BMI 18.5–24.99	Ref	Ref	Ref
BMI < 18.5	.572	.259–1.266	.168
BMI 25–29.9	.732	.451–1.190	.209
BMI ≥ 30	.715	.281–1.815	.480
Donor age (yrs)	1.009	.993–1.025	.277
History of DM	1.304	.830–2.049	.249
History of CKD	2.033	1.108–3.730	.**022**
History of IABP	5.063	2.176–11.780	**<**.**001**
History of ECMO	.654	.087–4.944	.681
Preop Ventilation	.347	.089–1.563	.178

The bold values indicated statistical significance.

Our results show that the recipient's BMI has no significant impact on post-transplantation survival. The underweight, overweight, and obese recipients were not a risk for follow-up mortality compared to the normal weight recipients on univariable and multivariable analysis. Some preoperative variables, such as recipient gender and diabetes status, were significantly different among the groups, but Cox regression analysis showed that those significant preoperative variables had no impact on follow-up mortality. Our results suggest that recipients should not be excluded from the HTx candidacy solely based on recipient BMI. In multivariable analysis, preoperative IABP use, history of CKD, and recipient age were independent risk factors for mortality. IABP use was associated with a five times while a history of CKD was associated with a two times increased risk of mortality. A one-year increase in recipient age was associated with a 3.4% increased risk of follow-up mortality. We combined the overweight and obese groups into a single group and performed the survival and regression analysis for three recipient BMI-based groups: Underweight, normal weight, and overweight/obese groups for better statistical power, but the conclusion remained unchanged. The KM survival curve and the Cox regression table can be found in the Supplementary Figure S1, Table S1, respectively.

## Discussion

Obesity has become a pandemic and has shown a significant association with the risk of heart failure (26). For every 1 unit increase in BMI, the incidence of heart failure increases by 5% in males and 7% in females (4). A large study of 190,672 persons by Khan et al. (27) evaluated the association of BMI with the incidence of cardiovascular disease morbidity and mortality. They found that being overweight and obese is associated with an increased risk of cardiovascular disease (CVD) compared to a normal BMI. Obesity was associated with a shorter life span, while overweight individuals had similar longevity compared to normal-weight individuals. Both obese and overweight patients lived a large proportion of their lives with CVD. In the subtype of CVD, obesity had the strongest association with heart failure. The HR for the first event of HF among middle-aged (40–59 years) overweight (BMI 25–29.9 kg/m^2^), obese (BMI 30–39.9 kg/m^2^), and morbidly obese (BMI ≥ 40 kg/m^2^) men was 1.22, 1.95, and 5.26 respectively, while for middle-aged overweight, obese, and morbidly obese women was 1.37, 2.28, and 4.32 respectively compared to normal weight individuals (BMI 18.5–24.9 kg/m^2^) (27).

Heart transplantation is still considered the best option for eligible end-stage heart failure patients (28). Some studies have identified obesity as a risk factor for post-HTx complications. A multicenter study by Bonet et al. (29) on multivariable Cox regression analysis found that obesity (BMI > 25 kg/m^2^) was significantly associated with long-term mortality (HR = 1.3, CI = 1.0–1.7, *P* = 0.02) after heart transplantation (29). Various other studies have linked higher BMI to worse heart transplantation outcomes, particularly in patients with significant obesity (17, 18, 23, 24).

Another study from the ISHLT registry by Healy et al. (30) evaluated the preoperative risk factors influencing early post-transplantation survival in patients bridged to heart transplantation with the continuous flow left ventricular assist device (CF-LVAD). They found that BMI is an independent risk factor for short-term mortality, with higher BMI associated with higher mortality rates. A study by Clerkin et al. (31) also reached the same conclusion that the survival of higher BMI patients bridged to transplant with CF-LVAD is lower after transplantation compared to normal-weight patients. It shows that the postoperative survival outcomes of obese patients are worse than those of normal BMI patients, but these results can't be generalized because this is a specific population bridged to HTx with CF-LVAD.

However, conflicting evidence exists. Some studies have suggested that high recipient BMI is not a risk factor for post-heart transplantation mortality. Russo et al. (22) used the UNOS database and divided patients into five BMI categories. They found no significant association between obesity (BMI 30–34.9 kg/m^2^) and long-term mortality in HTx recipients. Kocher et al. (32) also found no difference in survival in their study population, divided into four different BMI categories. Still, the groups were different in the incidence of wound complications, which required antibiotic therapy or surgery, with the highest in the group of BMI > 27 kg/m^2^. Weiss et al. (33) in their study of 27,002 patients from 1998–2007 concluded that patients with higher BMI waited longer and had lower likelihood of receiving a donor heart but did not experience increased mortality rates within one month, three months, or 1 year after HTx, while patients with a BMI ≥ 35 kg/m^2^ with UNOS status 1 had the lowest survival on wait list of 61% at 3 years (33). Our study included a small number of obese patients, and unfortunately, due to missing data on postoperative wound complications and the waiting time from listing to surgery, we were unable to assess these clinically relevant outcomes. The Canadian multicenter transplantation experience analyzed the association between multiple risk factors and early mortality. In multivariable logistic regression analyses, they found that BMI does not significantly influence one-month postoperative mortality (20). A multicenter study by Clark et al. (21) also found that baseline BMI did not affect survival. They believe that underweight patients can successfully undergo HTx and can achieve greater benefit from this procedure, and should not be excluded from HTx (21). Therefore, studies in the literature support both ideas, and further research should be conducted to clarify this concept.

Our study supports the notion that recipient BMI has no significant impact on post-transplantation survival, even after adjusting for confounders. Post-transplantation complications were also comparable between the groups, but these results can be influenced by baseline differences between the groups. The only statistically significant difference in post-HTx outcomes in our study was the use of postoperative IABP, which was higher in the obese (BMI ≥ 30 kg/m^2^) group compared to the other three groups. The number of patients with a BMI ≥ 30 kg/m^2^ was 36, and those with a BMI < 18.5 kg/m^2^ were 70 only, which is not a very large number, and may have influenced our results. In our study, only four patients had a BMI higher than 35 kg/m^2^, and the other 32 had a BMI of 30–35 kg/m^2^. It is very possible that the outcomes are not favorable, especially in patients with a BMI ≥ 35 kg/m^2^, which we don't deny based on our study results, and we limit our results to class I obesity only.

## Limitation

The results of this study should be evaluated in the light of its limitations. This is a retrospective observational study, and the effect of confounders, especially unmeasured confounders, can not be eliminated. The sample size in the two extreme BMI groups is relatively small, which can decrease statistical power, and the results should be interpreted cautiously. Thirty-two out of 36 obese patients belong to Class I obesity, and the results cannot be generalized to all obesity classes. Some missing outcomes data, such as cardiac allograft vasculopathy, wound complications, cardiac-related mortality, waiting time to transplantation, etc., limit the generalizability of this study. Multicenter, prospective studies with complete data, and a large sample size are required to refine the role of BMI in extreme subgroups.

## Conclusion

In our study, recipient BMI did not significantly impact post-heart transplantation survival after multivariable adjustment. While most non-survival postoperative complications were comparable between BMI groups in unadjusted analysis, obese recipients demonstrated a significantly higher incidence of postoperative IABP use. The preoperative IABP use, chronic kidney disease, and recipient age emerged as independent predictors for follow-up mortality. The preoperative IAPB use increased the risk of mortality by five times, while CKD increased the risk of mortality twice. A one-year increase in recipient age increased mortality risk by 3.4%. These findings suggest that BMI alone should not disqualify underweight and class I obese candidates from heart transplantation when other critical risk factors are accounted for in recipient selection.

## Data Availability

The original contributions presented in the study are included in the article/Supplementary Material, further inquiries can be directed to the corresponding authors.

## Editor's note

Hendrik Tevaearai Stahel edited the article in collaboration with Andreas Zuckermann, University of Vienna, Austria.
