# Correction: Impact of recipient body mass index on heart transplantation outcomes

**DOI:** 10.3389/fcvm.2025.1716429

**Published:** 2025-10-21

**Authors:** Frontiers Production Office

**Affiliations:** Frontiers Media SA, Lausanne, Switzerland

**Keywords:** heart transplantation, recipient BMI, survival outcomes, post-transplantation outcomes, obesity

The Editor's note was erroneously omitted. The Editor's note appears below.

**Editor's note**

Hendrik Tevaearai Stahel edited the article in collaboration with Andreas Zuckermann, University of Vienna, Austria.

The original article has been updated.

