# Supplementary material for: Impact of recipient body mass index on heart transplantation outcomes
Source: Front Cardiovasc Med. 2025 Sep 25;12:1573589. doi: 10.3389/fcvm.2025.1573589 (PMC12507866; doi:10.3389/fcvm.2025.1573589)
Supplement: Supplementary file 1 [file Datasheet1.pdf]

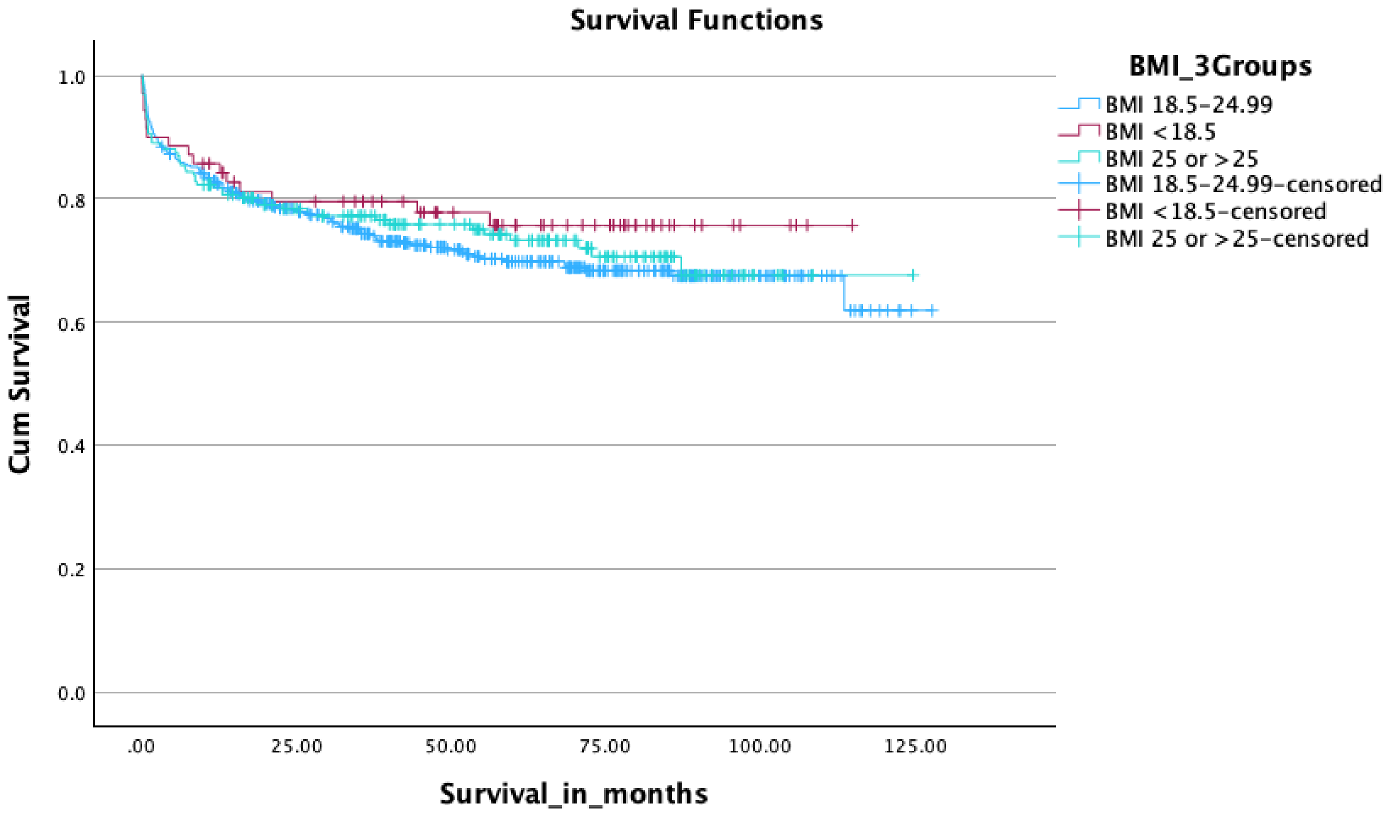

Supplementary Figure 1. The KM survival curves of the 3 recipient BMI groups after combining the overweight and obese groups into one (BMI ≥25) group. The log-rank p-value was .602.

Variables	Univariate analysis			Multivariate analysis		
	HR	95% CI	p-value	HR	95% CI	P value
Recipient age	1.028	1.015-1.041	**<.001**	1.034	1.016-1.052	**<.001**
Recipient gender (male)	.698	.506-.964	**.029**	.764	.487-1.199	.242
BMI 18.5-24.99 (kg/ m^2)	Ref	Ref	Ref	Ref	Ref	Ref
BMI <18.5	.775	.460-1.304	.336	.631	.290-1.376	.247
BMI ≥25	.926	.668-1.282	.643	.740	.475-1.152	.182

Donor age	1.018	1.005-1.031	**.006**	1.010	.994-1.026	.238
History of DM	1.523	.989-2.343	.056	1.348	.852-2.132	.203
History of CKD	2.24	1.312-3.823	**.003**	2.033	1.108-3.730	**.022**
History of IABP	3.747	1.830-7.670	**<.001**	5.063	2.176-11.780	**<.001**
History of ECMO	3.036	1.428-6.569	**.004**	.654	.088-4.877	.679
Preop Ventilation	2.523	1.325-4.803	**.005**	.347	.089-1.563	.178

Supplementary Table 1. Univariate and multivariate analysis after combining the overweight and obese groups in a single (BMI ≥25) group.

Note: A few minor fluctuations in values of non-significant variables were attributed to covariance adjustments and their elimination from the model after merging overweight and BMI groups, but did not alter the conclusion.
